# Quantitative brain T1 maps derived from T1-weighted MRI acquisitions: a proof-of-concept study

**DOI:** 10.1186/s41747-024-00517-2

**Published:** 2024-10-08

**Authors:** Audrey Lavielle, Noël Pinaud, Bei Zhang, Yannick Crémillieux

**Affiliations:** 1 Institut des Sciences Moléculaires, UMR5255, Université de Bordeaux, Talence, France; 2 Canon Medical Systems Europe, Zoetermeer, Netherlands

**Keywords:** Brain, Brain neoplasms, Contrast media, Magnetic resonance imaging, T1 mapping

## Abstract

**Background:**

Longitudinal T1 relaxation time is a key imaging biomarker. In addition, T1 values are modulated by the administration of T1 contrast agents used in patients with tumors and metastases. However, in clinical practice, dedicated T1 mapping sequences are often not included in brain MRI protocols. The aim of this study is to address the absence of dedicated T1 mapping sequences in imaging protocol by deriving T1 maps from standard T1-weighted sequences.

**Methods:**

A phantom, composed of 144 solutions of paramagnetic agents at different concentrations, was imaged with a three-dimensional (3D) T1-weighed turbo spin-echo (TSE) sequence designed for brain imaging. The relationship between the T1 values and the signal intensities was established using this phantom acquisition. T1 mapping derived from 3D T1-weighted TSE acquisitions in four healthy volunteers and one patient with brain metastases were established and compared to reference T1 mapping technique. The concentration of Gd-based contrast agents in brain metastases were assessed from the derived T1 maps.

**Results:**

Based on the phantom acquisition, the relationship between T1 values and signal intensity (SI) was found equal to T1 = 0.35 × SI^−^^1.11^ (*R*^2^ = 0.97). TSE-derived T1 values measured in white matter and gray matter in healthy volunteers were equal to 0.997 ± 0.096 s and 1.358 ± 0.056 s (mean ± standard deviation), respectively. Mean Gd^3+^ concentration value in brain metastases was 94.7 ± 30.0 μM.

**Conclusion:**

The *in vivo* results support the relevance of the phantom-based approach: brain T1 maps can be derived from T1-weighted acquisitions.

**Relevance statement:**

High-resolution brain T1 maps can be generated, and contrast agent concentration can be quantified and imaged in brain metastases using routine 3D T1-weighted TSE acquisitions.

**Key Points:**

- Quantitative T1 mapping adds significant value to MRI diagnostics.
- T1 measurement sequences are rarely included in routine protocols.
- T1 mapping and concentration of contrast agents can be derived from routine standard scans.
- The diagnostic value of MRI can be improved without additional scan time.

**Graphical Abstract:**

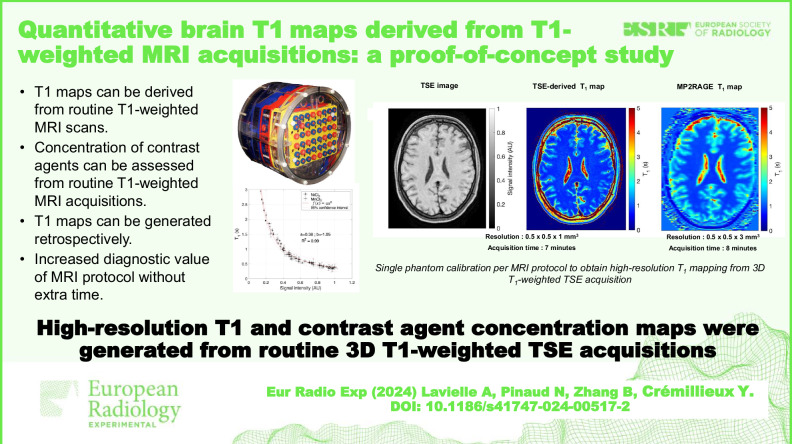

## Background

The longitudinal T1 relaxation time of water protons *in vivo* is obviously one of the most important parameters in magnetic resonance imaging (MRI). It enables the generation of contrasts between different tissues using T1-weighted imaging sequences, which are typically fast, have anatomical-quality spatial resolution and form part of the basis of imaging protocols used in clinical practice. Moreover, T1 values are well-known imaging biomarkers for many pathologies, such as multiple sclerosis and Parkinson’s disease [1, 2]. Finally, T1 maps can be used for various applications, such as arterial spin labeling [3, 4] or studies on children with neurological developmental delays [5].

In addition, contrast agents are essential in clinical MRI due to their ability to selectively modify T1 values in healthy or pathological tissues, or in biological compartments such as the vascular system. The use of the T1 contrast agents is therefore ubiquitous, for example, in the diagnosis and prognosis of cancer and for disease and therapy monitoring. In the vast majority of cases where the aim is solely to enhance and delineate the tumor, imaging the distribution of contrast agent in tumors can remain qualitative [6]. For certain key applications, however, such as dynamic contrast-enhanced perfusion imaging, a quantitative assessment of the changes in T1 values due to the administered contrast agent is required to obtain maps of blood volume, blood flow, or mean transit time in tumors [7]. Similarly, in the case of theranostic agents it is important to quantify the changes in T1 values and to assess the concentration of the T1 agents in the tissues [8, 9].

Thus, quantitative parametric T1 maps are a relevant issue. Numerous sequences are available to generate quantitative T1 maps in patients [10–14]. However, in clinical practice, these sequences are rarely incorporated into clinical protocols due to limited access time to MRI, availability of sequences, or protocol duration limitations. As a result, the radiologists, clinicians, or scientists must often rely only on T1-weighted acquisitions. Ideally, therefore, deriving T1 maps from standard T1-weighted acquisitions would be very valuable.

In this study, we propose a versatile approach to T1 mapping based on the use of a calibration phantom to establish a correspondence between image intensity in T1-weighted acquisition and T1 values. To assess the feasibility and robustness of this approach, we applied it to MRI datasets acquired with three-dimensional (3D) T1-weighted turbo spin-echo (TSE) sequences on healthy volunteers. We also applied this approach to datasets acquired from a patient with multiple brain metastases before and after administration of Gd-based nanoparticles (pre- and post-T1 maps were used to assess the concentration of nanoparticles in the brain metastases). In this article, the proposed approach will be referred to as CASual Phantom for Easy Relaxometry (CASPER).

## Methods

### Phantom composition and characterization

A home-made phantom composed of 144 2-mL tubes containing solutions of either MnCl_2_, NiCl_2_ or CuSO_4_ was used to determine a relation between the signal intensity (SI) from 3D T1-weighted TSE images and the longitudinal relaxation time T1. The phantom was composed of 48 tubes of each solution, and the tubes were arranged on 3-stacked square plate support corresponding to a total volume of 9.9 × 10 × 11.5 cm^3^.

**Fig. 1:**
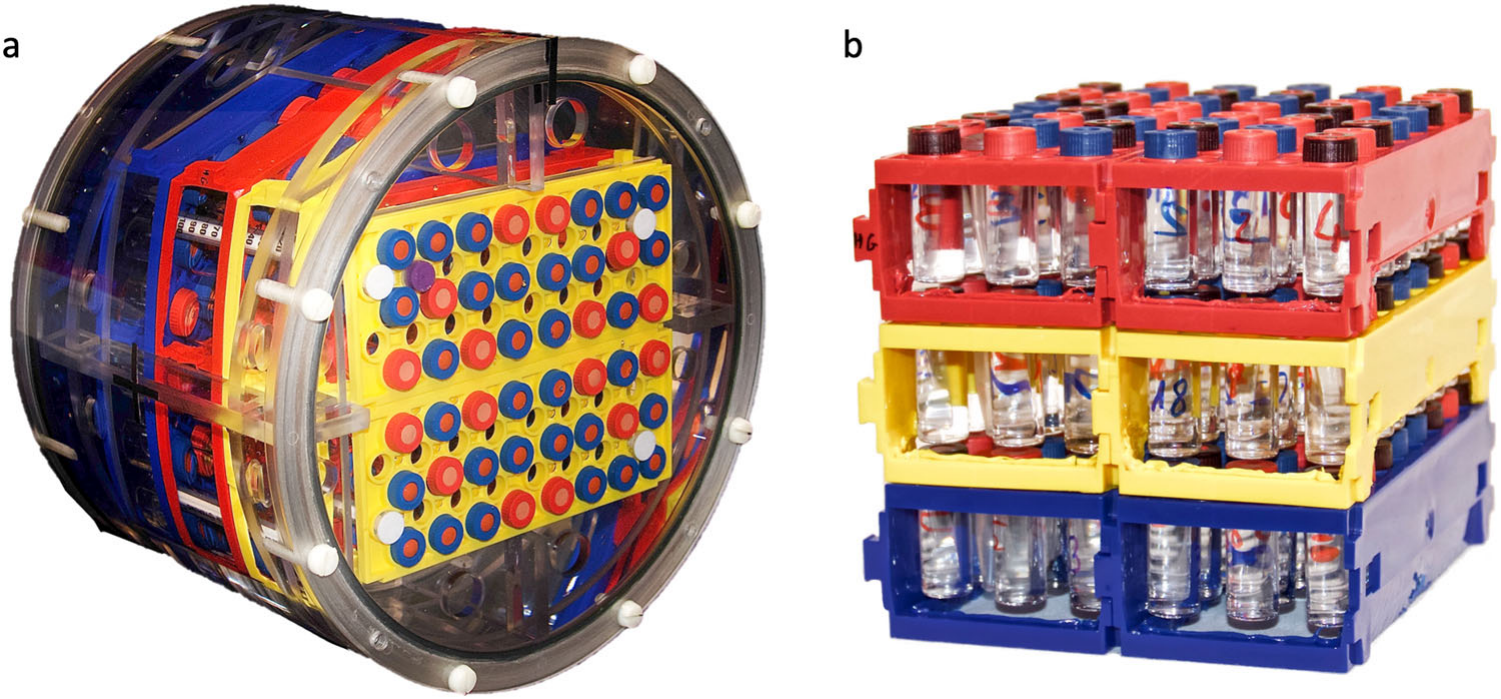
Home-made phantom. **a** The phantom is composed of 144 tubes containing solutions of either MnCl_2_, NiCl_2_, or CuSO_4_, distributed over 3 stacked square plates and immersed in a NaCl saline solution. **b** Support and the tubes out of the container

The concentration of the MnCl_2_ and NiCl_2_ in the tubes ranged between 0.002 and 0.472 mM in 0.01 mM increments, and 0.030 and 4.730 mM in 0.1 mM increments, respectively. The concentration of CuSO_4_ in the tubes was fixed to 1 mM. The concentration ranges of these paramagnetic ions were chosen on the basis of their longitudinal relaxivity r_1_ at 3 T (r_1_ = 6.4 mM^−^^1^.s^−^^1^ for MnCl_2_ and r_1_ = 0.6 mM^−^^1^.s^−^^1^ for NiCl_2_) [15] in order to obtain a range of T1 values representative of those found *in vivo*. All tubes and support were immersed in a container filled with a 9 g/L NaCl saline solution, as shown in Fig. 1.

### MRI experiments

#### The experiments were performed on 3-T clinical scanners

A 3-T Vantage Galan ZGO scanner (Canon Medical Systems Corporation, Otawara, Japan) was used to perform 3D T1-weighted TSE acquisitions (“3D MVOX” on this equipment) on the phantom and on healthy volunteers, using the following parameters: echo time 24 ms; repetition time 600 ms; number of echoes per radiofrequency (RF) pulse excitation 15; image size 416 × 416 × 340; field of view: 208 × 208 × 340 mm^3^; spatial resolution: 0.5 × 0.5 × 1 mm^3^; total acquisition time: 5:53 min:s. A bias field correction related to RF inhomogeneities was applied with the FMRIB Software Library and the FAST tool (version 6.0.6; FMRIB, Oxford, UK). The same whole-body coil for RF excitation and 32-channel head coil for reception were used for phantom and volunteer acquisition.

A 3-T Achieva scanner (Philips Healthcare, Best, The Netherlands) was used to perform 3D T1-weighted TSE acquisitions (“VISTA” on this equipment) on the phantom and a patient with brain metastases, using the following parameters: echo time 30 ms; repetition time 600 ms; number of echoes per RF pulse excitation 22; image size 480 × 480 × 350; field of view: 240 × 240 × 175 mm^3^; spatial resolution: 0.5 × 0.5 × 0.5 mm^3^; total acquisition time: 4:57 min:s. A “constant level appearance correction” was automatically applied to the T1-weighted TSE images by this scanner. This correction was used to achieve homogeneity correction by using coil sensitivity maps acquired in a reference scan. The same whole-body coil for RF excitation and 32-channel head coil for reception were used for phantom and patient acquisition.

##### Phantom acquisitions

The T1 values of the 144 tubes composing the phantom were measured using a magnetization prepared 2 rapid acquisition gradient-echoes (MP2RAGE) sequence [14] on the 3-T Canon scanner, with the following parameters: echo time 3.3 ms; repetition time 7.5 ms; inversion time 655/3,300 ms; image size: 384 × 384 × 50; field of view: 190 × 190 × 150 mm^3^; spatial resolution: 0.5 × 0.5 × 3.0 mm^3^; total acquisition time: 7:42 min:s. 3D T1-weighted TSE images of the phantom were acquired on the two scanners. These acquisitions were repeated with the phantom rotated 45° from its initial position to investigate the sensitivity of SI to changes in tube position. CuSO_4_ tubes with the same concentration of 1 mM uniformly distributed throughout the phantom were used to assess the impact of inhomogeneities of the B_1_ RF field on the SI of the TSE sequence. To this end, variations in the amplitude of the signal as a function of the position of the CuSO_4_ tube in the coil were measured.

##### Acquisition in healthy volunteers

Four healthy volunteers, two men and two women (aged 52, 58, 25, and 49 years, respectively), participated in the study. T1-weighted TSE images of the volunteers were acquired on the same 3-T Canon scanner that was used for the phantom acquisition, using the same whole-body RF excitation coil and receive head coil and identical parameters of the 3D T1-weighted TSE sequence. MP2RAGE acquisitions were also performed using the same parameters as for phantom acquisitions.

##### Acquisition in the brain metastasis patient

The patient with brain metastases included in this study underwent the same protocol as the one reported by Verry et al [8, 9]. The patient was administered an intravenous solution of Gd-based nanoparticles consisting of a polysiloxane network surrounded by cyclic gadolinium ligands, which are derivatives of 1,4,7,10-tetraazacyclododecane-1,4,7,10-tetraacetic acid (DOTA) covalently grafted to the polysiloxane matrix [16]. The longitudinal relaxivity r_1_ of the nanoparticles at 3 T, obtained from saline solution at room temperature, was measured to be 7.9 mM^−^^1^.s^−^^1^ per Gd^3+^ ion.

T1-weighted TSE images were obtained before the intravenous injection and approximately 1 h after the injection of nanoparticles at 100 mg/kg. The 3D T1-weighted TSE images of the patient included in this study were acquired on the same 3-T Philips scanner that was used for the phantom acquisition, using the same whole-body RF excitation coil and receive head coil and identical parameters of the 3D T1-weighted TSE sequence.

### Computation of T1 maps

As a first step, the T1 values in the tubes obtained with the MP2RAGE measurements were plotted as a function of the T1-weighted TSE SI for each tube composing the phantom.

The curve was fitted with a power function:

1 $${{{\rm{T}}}}1={{{\rm{a}}}}\times {{{{\rm{SI}}}}}^{{{{\rm{b}}}}}$$

with a and b being two parameters determined using a power regression.

To account for variations in parameter “a” from one TSE acquisition to another, for example, due to changes in signal amplification, the amplitude of the T1-weighted TSE signal in a region of interest (ROI) with a known T1 value, such as one of the phantom tubes or tissue with well-established nuclear magnetic resonance properties, was measured to provide a pair of values (image intensity, T1). The pair of values from this scaling ROI was used to determine the value of parameter “a” in Eq. 1 for a given T1-weighted TSE acquisition.

For 3D T1-weighted TSE acquisition on phantom, the scaling ROI was positioned in a CuSO_4_ tube in the center of the phantom. For the acquisition in healthy volunteers, the ROI was located in the occipital white matter (WM). The T1 value of WM was set to 0.900 s according to the values reported in the literature [17]. For the acquisition in the patient with brain metastases, the ROI was located in the occipital gray matter (GM). The T1 value of GM was set to 1.350 s according to the values reported in the literature [17]. The variations of proton density present *in vivo* in GM, WM, and cerebrospinal fluid (CSF) were taken into account before computing the T1 maps. The proton density values in WM, GM, and CSF used in this study were set equal respectively to 66%, 83%, and 100% of proton density of pure water at 37 °C as reported in the literature [17].

Mean signal intensities were measured in three ROIs located in occipital WM, occipital GM and the right lateral ventricle. Based on the intensity of the signal relative to the values in these three ROIs, each voxel in the TSE images was then assigned a proton density value by linear interpolation [18]. The ratio between the proton density value in each voxel and the proton density value in the scaling ROI was calculated to generate a proton density correction map. The T1-weighted TSE SI in each voxel was then divided by the proton density correction map.

The SI of each voxel of the proton-density corrected TSE 3D dataset was then assigned a T1 value using the power function with parameters a and b. Using this one-to-one bijective correspondence for each voxel of the T1-weighted TSE images, a 3D map of T1 values was then generated.

The summary of all the necessary steps to compute the T1 maps can be summarized as follows:

1. measurements of the T1 values of the phantom tubes using a reference T1 mapping sequence, namely the MP2RAGE sequence in this study;
2. acquisition of 3D T1-weighted TSE images of the phantom;
3. plot of the T1 value in each tube as a function of its TSE SI and determination of the parameters a and b of the power function T1 = a × SI^b^.
4. acquisition of 3D T1-weighted TSE images on a subject.
5. measurement of the SI in a scaling ROI with a known T1 value to determine the parameter “a” of the power function.
6. measurements of SIs in ROIs in WM, GM, and CSF to generate a proton density map based on proton density values from the literature;
7. generation of a proton-density corrected TSE 3D dataset;
8. computation of the 3D T1 map from the proton-density corrected TSE 3D dataset using the power function T1 = a × SI^b^.

### Computation of concentration maps

To quantify the concentration of Gd-based nanoparticles *in vivo*, T1 maps derived from T1-weighted TSE acquisitions performed before and after the administration of the nanoparticles were used. Image registration was performed between the pre- and post-contrast TSE images to correct for changes in the patient position. In each voxel, the concentration of the contrast agent was computed using the following formula:

2 $$\frac{1}{{T}_{1,{post}}}=\,\frac{1}{{T}_{1,{pre}}}+{r}_{1}C$$

where ${T}_{1,{pre}}$ and ${T}_{1,{post}}$ are the T1 values (in second unit) before and after the administration of the contrast agent, and ${r}_{1}$ the longitudinal relaxivity of the contrast agents (in mM^−^^1^_._s^−^^1^).

ROIs were manually drawn in all metastases with a volume greater than 125 mm^3^ and in healthy tissue: occipital and frontal WM, frontal GM, and CSF. The mean and standard deviation of the T1 and concentration values were computed for each of these ROIs.

### Computation, image analysis and statistical analysis

All computations, curve fitting, data processing, and image display presented in this study were conducted using MATLAB software (version 9.12; The MathWorks, Natick, MA, USA). For image registration and ROIs delineation, Olea SDK software (version 1.5.3; Olea Medical, La Ciotat, France) was used.

As mentioned above, a power regression was used to establish the relationship between the MP2RAGE-derived reference T1 values of each tube and the T1-weighted TSE SI in these tubes. Linear regression without intercept was used to evaluate changes between TSE-derived T1 values with the phantom in its initial position and rotated by 45°.

Linear regression without intercept was used to characterize the relationship between TSE-derived T1 values and MP2RAGE-derived reference T1 values for all the tubes composing the phantom. Coefficient of determination *R*^2^ was used to assess how well the model fit the data.

Data are presented as the arithmetic mean value ± standard deviation.

## Results

### Phantom experiments

**Fig. 2:**
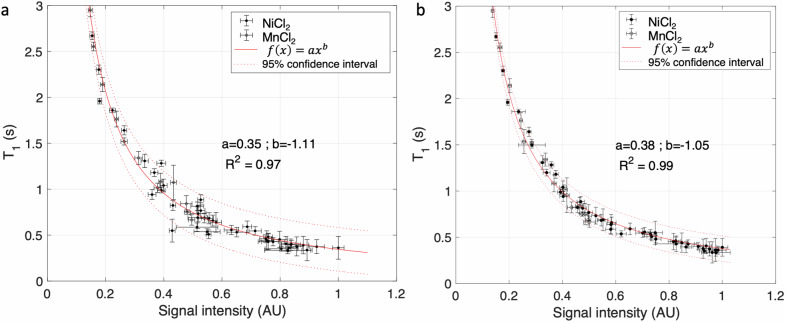
T1 value (obtained with the MP2RAGE measurements) as a function of signal intensity in T1-weighted TSE image for NiCl_2_ (black circles) and MnCl_2_ (white circles) tubes from the phantom obtained on the Canon scanner (**a**) and the Philips scanner (**b**). The red curve represents a power regression, and dotted red curves represent the 95% confidence interval. Vertical and horizontal error bars correspond to standard deviations on the T1 values and signal intensity values measured in regions of interest in the tubes. MP2RAGE, Magnetization prepared 2 rapid acquisition gradient-echoes; TSE, Turbo spin-echo

The T1 value of the NiCl_2_ and MnCl_2_ tubes plotted as a function of the T1-weighted TSE SI measured in ROIs in each tube on the two scanners are shown in Fig. 2. Due to the r_2_ relaxivity induced by Mn^2+^ ions, MnCl_2_ tubes with T1 relaxation times shorter than 0.5 s were excluded from the calibration curve.

For the acquisition performed on the Canon scanner, the parameters a and b of the Eq. 1 power function were a = 0.35 and b = -1.11, with a coefficient of determination *R*^2^ equal to 0.97. The power function was also established separately for NiCl_2_ tubes only and for MnCl_2_ tubes only. The a and b coefficients of the power function were found equal to 0.34 and -1.13 and to 0.38 and -1.06 for NiCl_2_ and MnCl_2_, respectively. *R*^2^ was equal to 0.96 and 0.99 for NiCl_2_ and MnCl_2_ tubes, respectively. For the acquisition performed on the Philips scanner, the parameters a and b of the Eq. 1 power function were a = 0.38 and b = -1.05, with a coefficient of determination *R*^2^ equal to 0.99. The power function was also established separately for NiCl_2_ tubes only and for MnCl_2_ tubes only. The a and b coefficients of the power function were 0.38 and -1.05 and 0.37 and -1.06 for NiCl_2_ and MnCl_2_ tubes, respectively, with *R*^2^ equal to 0.99 in both cases. The power functions obtained from the power regression between T1 value and T1-weighted TSE SI on two scanners are plotted in Fig. 2.

**Fig. 3:**
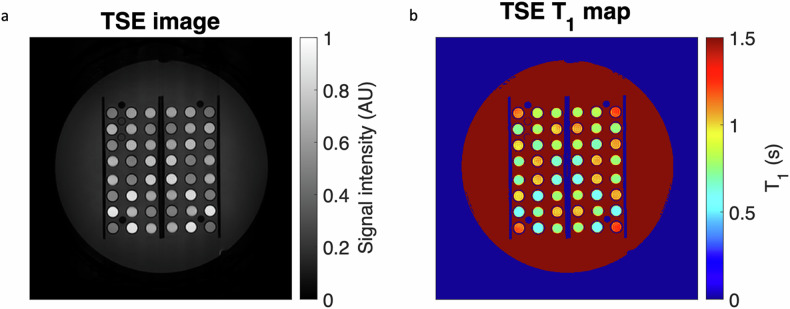
**a** TSE image corresponding to the phantom central plate. **b** Corresponding T1 map derived from the TSE acquisition. TSE, Turbo spin-echo

**Fig. 4:**
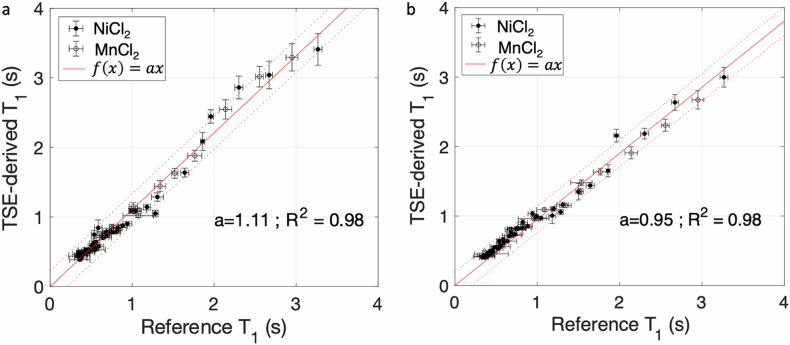
TSE-derived T1 values *versus* reference T1 values obtained from MP2RAGE acquisitions for NiCl_2_ (black circles) and MnCl_2_ (white circles) tubes from the phantom obtained on the Canon scanner (**a**) and the Philips scanner (**b**). The red curve represents a linear regression. Vertical and horizontal error bars correspond to standard deviations on the T1 values measured in regions of interest in the tubes. MP2RAGE, Magnetization prepared 2 rapid acquisition gradient-echoes; TSE, Turbo spin-echo

Examples of T1-weighted TSE image and T1 map derived from Eq. 1 for the central plate of the phantom are shown in Fig. 3. The TSE-derived T1 values measured in ROIs positioned in NiCl_2_ and MnCl_2_ tubes are plotted in Fig. 4 as a function of the reference MP2RAGE-derived T1 values for the two scanners. The plot and linear regression showed an excellent match between the T1 measurements obtained with the two techniques for T1 values between 0.300 and 3.000 s, the slope was equal to 0.95 (*R*^2^ = 0.98) on the Philips scanner, and the slope was equal to 1.11 (*R*^2^ = 0.98) on the Canon scanner.

An excellent correlation was observed between the T1 values on the T1-weighted TSE images with the phantom in its initial position and rotated by 45°, the slope of the linear regression was 1.01 (*R*^2^ = 0.99).

**Fig. 5:**
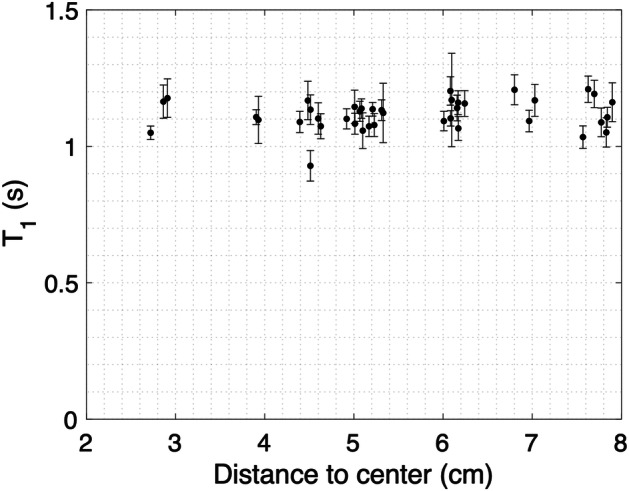
TSE-derived T1 values as a function of distance to the center of the phantom for CuSO_4_ tubes. Vertical error bars correspond to standard deviations on the T1 values. TSE, Turbo spin-echo

TSE-derived T1 values in CuSO_4_ tubes with identical concentration as a function of distance from the center of the phantom are shown in Fig. 5. The mean MP2RAGE-derived T1 value of these solutions was measured equal to 1.065 s. Considering CuSO_4_ tubes within 8 cm from the center, their TSE-derived T1 value was measured close to the reference T1 value, with a mean TSE-derived T1 value and standard deviation of 1.117 ± 0.055 s.

### T1 measurements in healthy volunteers

**Fig. 6:**
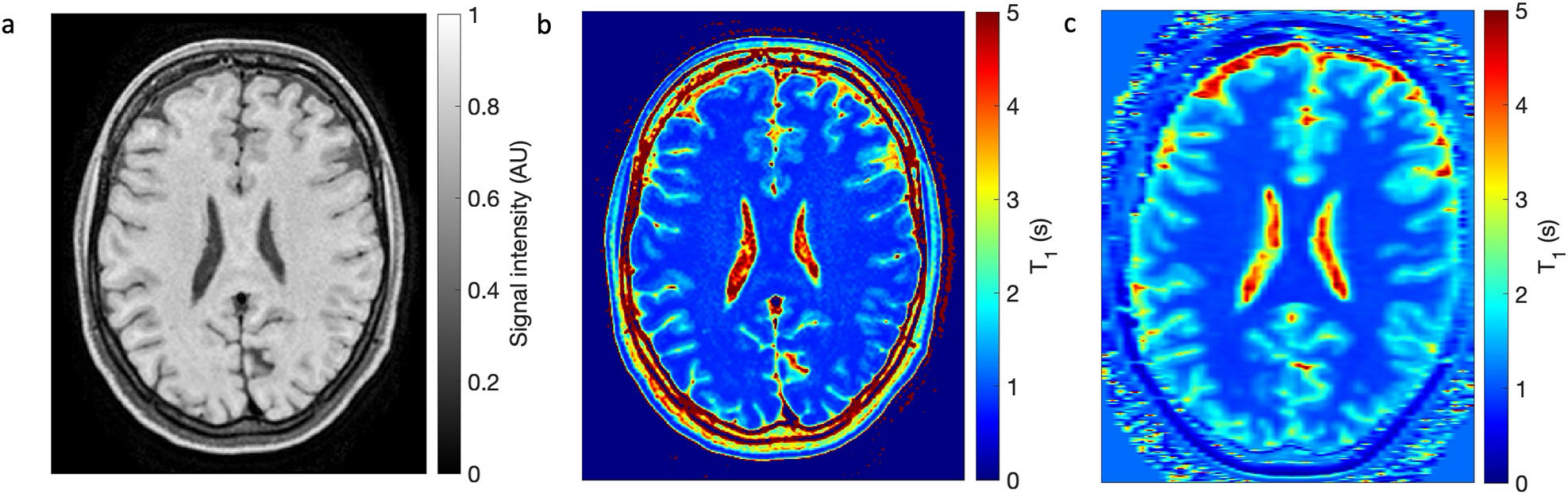
**a** Three-dimensional T1-weighted TSE image acquired from healthy volunteer #1. **b** The T1 map derived from the TSE image. **c** a MP2RAGE T1 map obtained at the same position. MP2RAGE, Magnetization prepared 2 rapid acquisition gradient-echoes; TSE, Turbo spin-echo

An example of a 3D T1-weighted TSE image from a volunteer is shown in Fig. 6a. The corresponding T1 map derived from the SI of the TSE image using Eq. 1 is shown in Fig. 6b. The spatial resolution of the T1 map is the same as that of the TSE image, namely 0.5 × 0.5 × 1 mm^3^. In Fig. 6c, for comparison, the T1 map obtained from the MP2RAGE acquisition is shown, with a spatial resolution of 0.5 × 0.5 × 3.0 mm^3^.

Table 1 presents T1 values in several brain regions derived from the TSE images for the four volunteers. The mean TSE-derived T1 values measured in WM regions, GM regions and CSF were found in the range of values reported in the literature and similar to those obtained with the MP2RAGE sequence, with the exception of CSF value obtained with the MP2RAGE sequence [19].

**Table 1:** Mean and standard deviation of T1 values for four healthy volunteers obtained from three-dimensional T1-weighted TSE acquisitions in brain regions: occipital and frontal WM, frontal GM, and CSF

	TSE-derived T_1_ (s)	T_1_ literature value (s) [17, 19]	MP2RAGE-derived T_1_ (s)
Occipital WM	0.997 ± 0.096	0.700–1.000	0.958 ± 0.082
Frontal GM	1.408 ± 0.130	1.000–1.600	1.440 ± 0.170
Occipital GM	1.358 ± 0.056		1.403 ± 0.150
CSF	4.660 ± 0.354	3.800–5.000	2.792 ± 1.499

Data are given as mean ± standard deviation

*CSF* Cerebrospinal fluid, *GM* Gray matter, *MP2RAGE* Magnetization prepared 2 rapid acquisition gradient-echoes, *TSE* Turbo spin-echo, *WM* White matter

### T1 measurement and Gd^3+^ concentration in a patient with brain metastases

**Fig. 7:**
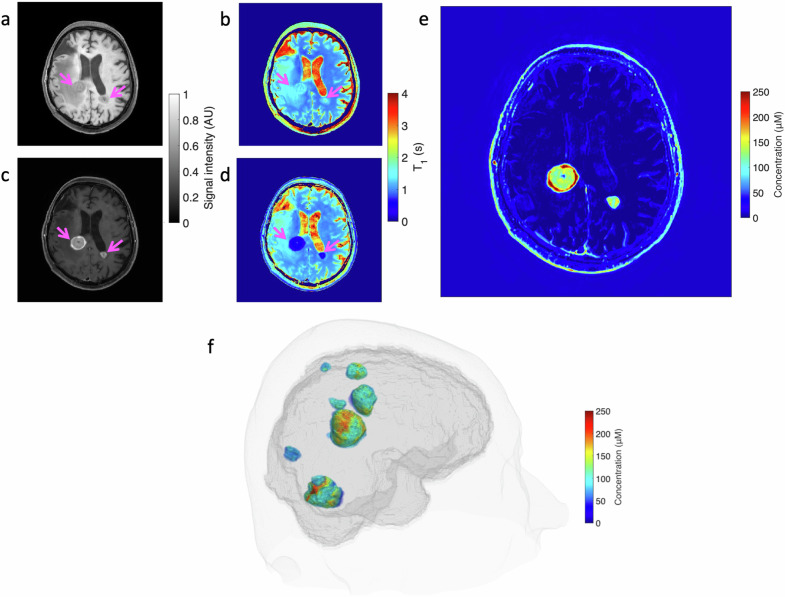
**a** 3D T1-weighted TSE image acquired from the patient with brain metastases. **b** Corresponding T1 map before the administration of the Gd-based nanoparticles. **c** 3D T1-weighted TSE image. **d** Corresponding T1 map derived from TSE images 1 h after the administration of the gadolinium-based nanoparticles. **a**–**d** Pink arrows are pointing at the two brain metastases. **e** Gd^3+^ concentration map derived from the pre- and post-injection T1 maps. **f** 3D whole-brain visualization of Gd^3+^ concentrations into metastases obtained using 3D TSE T1-weighted acquisitions in the patient. 3D, Three-dimensional; TSE, Turbo spin-echo

Examples of T1-weighted TSE images acquired in the patient pre- and post-injection of gadolinium-based nanoparticles and the corresponding derived T1 maps are shown in Fig. 7. The uptake of gadolinium-based nanoparticles in the TSE images was clearly visible in the two brain metastases. Similarly, changes in T1 values in metastases following the administration of the theranostic agent were readily visible 1 h after the injection.

The mean and standard deviation of Gd^3+^ concentrations, computed using Eq. 2, in the left and right metastases were equal to 143.1 ± 67.4 and 85.3 ± 48.1 μM, respectively. The mean and standard deviation of the concentration of Gd^3+^ ion measured in all the metastases (*n* = 8) with a volume greater than 125 mm^3^ was equal to 94.7 ± 30.0 μM.

In regions not identified as brain metastases, there was no evidence of variations in T1 values before and after injection of gadolinium-based nanoparticles. T1 values varied by -5% in the occipital WM, -3% in the frontal WM and +7% in the CSF. The Gd^3+^ concentration map shown in Fig. 7e illustrates the selective uptake of the nanoparticles in the metastases. Some artefactual concentration values due to imperfection in the image registration and small non-rigid displacements are noticeable at the interface between brain structures. A 3D whole-brain visualization of Gd^3+^ concentrations highlighting all the metastases of the patient is presented in Fig. 7f.

## Discussion

The paramagnetic agents used for the phantom, in particular NiCl_2_, were selected for their good stability over time and low-temperature dependence [20]. Stability over time ensures that the T1 values in the tubes remain in the same range and that the phantom can be stored and used weeks or months later without significant change in T1 values. In any case, each time the phantom is reused to generate a T1 value *versus* signal amplitude curve, it is preferable to remeasure the T1 values in the tubes with a reference sequence such as MP2RAGE. Temperature stability is not an issue as long as the phantom T1-weighted TSE acquisition and the T1 measurements of the solutions are performed under the same ambient temperature conditions.

The different concentrations of these paramagnetic agents in the tubes were chosen to provide T1 values between 0.4 and 3 s, a range of T1 values representative of those found in healthy brain tissue or tumor tissue with or without the presence of contrast agents. For this range of T1 values, the relationship between the SI of the T1-weighted TSE sequence and the T1 values was accurately established. In fact, for both scanners, a relationship very close to linearity was measured between the relaxation rate R_1_ = 1/T1 and the SI, as might be expected for a T1-weighted sequence.

To assess the sensitivity of the T1-weighted TSE sequence to T_2_ relaxation, the plot of T1 values *versus* signal amplitude was evaluated separately for NiCl_2_ and MnCl_2_ tubes. The r_2_/r_1_ ratio of the two paramagnetic cations differs markedly from each other. At 3 T, this ratio is equal to 1.4 and 16.9 for Ni^2+^ and Mn^2+^ respectively [15]. Despite this difference of more than one order of magnitude, the results of the power regression were found to be similar for the two types of contrast agent. These results indicate that the impact of T_2_ relaxation on signal amplitude can be neglected to a first approximation, as desirable for a T1-weighted sequence.

The excellent matching between the TSE-derived T1 values and the MP2RAGE reference T1 values confirms the relevance and robustness of the approach *in vitro* on contrast agent solutions.

To assess the impact of inhomogeneities of the B_1_ RF field on the SI of the TSE sequence and hence on T1 measurements, CuSO_4_ solutions with the same concentration, uniformly distributed throughout the phantom, were used. The T1 values obtained on these CuSO_4_ solutions indicate that reliable measurements can be achieved within a sphere of 16 cm in diameter. The homogeneity of the RF B_1_ field (transmit and receive) obviously varies from one scanner to another, depending on the hardware used (receive coil in particular) and the software solutions implemented. For the two scanners used in this study, it can be concluded that the impact of B_1_ variations on the measurement of T1 values is negligible over a volume similar to that of the brain. This finding is confirmed by the excellent correspondence between the SI values, and hence the T1 values, measured before and after rotation of the phantom by 45°.

Mapping T1 values in volunteers or patients requires, wherever possible, taking into account variations in tissue proton density affecting the amplitude of the TSE signal. In this study, proton density correction was performed by considering three proton density values applied to CSF, GM and WM. The relevance and the implementation of this proton density correction approach had already been demonstrated in a previous study [18]. The results obtained in four healthy volunteers show that this correction produces T1 values similar to those obtained with the MP2RAGE sequence and consistent with those reported in the literature in the distant occipital and frontal GM and WM regions.

In neuro-oncological MRI, and especially for brain metastasis imaging, the 3D T1-weighted TSE sequence, used in association with T1 contrast agent administration, has established itself as the reference sequence [21–23]. As shown by the T1 *versus* SI relationship obtained on the phantom, this sequence provides outstandingly pure T1 weighting with virtually no T2 weighting.

3D T1-weighted TSE images obtained 1 h after Gd-based nanoparticle administration reveal the uptake of these Gd-based agents in the metastases. This nanoparticle uptake was previously observed in magnetization prepared rapid gradient-echo (MPRAGE) T1-weighted images acquired in a clinical trial on the same patient population with multiple brain metastases [8, 9]. For the computation of T1 relaxation times in brain metastases of the patient, the unknown value of proton density in metastases was set equal to that of GM considered as a reasonable approximation.

Nanoparticle concentrations in metastases, assessed by the relationship between T1 values, nanoparticles concentration and relaxivity, were of the same order of magnitude as those obtained previously using MPRAGE-based T1 images and the same dose of injected nanoparticles [8, 18]. Similarly, there were no noticeable differences in T1 values in GM and WM tissues before and 1 h after nanoparticle injection. These results on T1 values are similar to those obtained in a previous study [18] and indicate a concentration of Gd-based nanoparticles below the threshold of detection by MRI in healthy brain tissue.

As already mentioned, in many clinical protocols and in particular in the one from which the acquisitions on the patient were obtained, sequences for measuring T1 relaxation times are not included. The proposed CASPER approach can overcome the lack of parametric T1 acquisition sequences in a clinical protocol and allow post-acquisition generation of T1 maps and Gd^3+^ concentration maps from clinical T1-weighted acquisitions. As demonstrated by the results obtained in volunteers and a patient in this proof-of-concept study, it is possible to generate T1 maps in subjects using the CASPER approach from a single 3D T1-weighted TSE acquisition. The use of this sequence, which is particularly well suited for the visualization of brain metastases and multiple sclerosis lesions [21, 24], allows the generation of T1 maps with the same spatial resolution as images obtained with the 3D T1-weighted TSE sequence. In this study, the spatial resolution was 0.5 × 0.5 × 1 mm^3^ for healthy volunteers and 0.5 × 0.5 × 0.5 mm^3^ for the patient. In comparison, the MP2RAGE T1 mapping in volunteers requires 35% more time for acquisition with a lower spatial resolution of 0.5 × 0.5 × 3 mm^3^. The CASPER approach can thus effectively overcome the absence of parametric T1 acquisition sequences in a clinical protocol, or can alternatively reduce protocol duration by avoiding acquisitions dedicated to T1 measurement. In addition to neuro-oncology, the approach could be used for other brain MRI indications where T1 values are known to be biomarkers of brain pathology [1, 24, 25].

In contrast to a previously reported T1 mapping approach, which required the analytical expression of the SI [18], the CASPER approach can be applied to T1-weighted acquisitions where this analytical expression is unknown, as is the case for 3D T1-weighted TSE. In principle, the use of this approach could be extended to other types of sequences for which strong T1 weighting exists, such as MPRAGE or volumetric interpolated breath-hold examination (VIBE) sequences, making this approach a versatile and generic T1 mapping technique.

One drawback of the approach is that it requires phantom calibration for a given configuration of the scanner, set of RF coils and 3D T1-weighted TSE sequence parameters used. However, once this calibration with the phantom has been carried out, the power function obtained can be used for all patients included in the protocol. Of note, the phantom calibration and the acquisitions on subjects were carried out several months apart in this study. T1 maps derived from T1-weighted 3D TSE acquisitions can therefore be generated retrospectively, provided the phantom can be imaged with the same sequence parameters and experimental setup. Although the phantom calibration procedure seems desirable for each scanner or sequence configuration, it is interesting to note that the results obtained for the power function parameters were very similar for the two scanners from different manufacturers.

The phantom used in this study comprised a large number of solutions, 144 tubes in total, with three different types of paramagnetic agents. As a proof-of-concept study, it was essential to have a sufficiently large number of points to establish as accurately as possible the function that relates T1 values to SI. To assess the sensitivity of the T1-weighted TSE sequence to T_2_ relaxation time values, both Ni^2+^ and Mn^2+^ were used in solutions. Finally, CuSO_4_ was chosen as a reference solution to evaluate the effect of B_1_ RF field inhomogeneities. In light of the results obtained, it seems possible to significantly reduce the number of solutions necessary for future applications. For example, the calibration procedure could be limited to the use of about twenty NiCl_2_ solutions with concentrations covering the T1 values *in vivo*. Because of its better stability over time, NiCl_2_ solutions of identical concentration could advantageously replace the CuSO_4_ solutions. With proper positioning of these identical solutions, a total of 15 tubes, 5 per plate, may be sufficient.

One of the limitations of this approach is the dependence of the T1-weighted 3D TSE signal on proton density. In healthy brain tissue, the effect of proton density can be largely compensated using reference proton density values available in the literature. In brain metastases, the proton density is not known a priori, and it is necessary to assign a proton density value with a certain margin of error. In the present study, the proton density of the surrounding GM was chosen as the most plausible proton density value for brain metastases. This type of proton density correction approach has been used previously for T1 mapping and Gd^3+^ concentration measurements in brain metastases [18]. Similarly, the proton density value in multiple sclerosis lesions could be reasonably approximated by the proton density value in the surrounding WM tissue.

For the determination of the concentration of T1 contrast agents in metastases, one underlying assumption is that the relaxivity of these agents *in vitro* and *in vivo* is the same. This assumption introduces uncertainty into the absolute concentration measured *in vivo* for these agents. However, despite these uncertainties, an assessment of relative concentration values is still ensured and provides the information needed to evaluate the uptake of contrast agents in tumors or metastases.

In conclusion, we presented here an approach designed to derive quantitative brain T1 mapping in subjects from a single 3D T1-weighted TSE acquisition. We showed as well that this T1 measurement technique can be used in patients with multiple brain metastases for assessing T1 changes following the administration of Gd-based contrast agents and for measuring the concentration of these contrast agents in metastases.

## Supplementary information

**Additional file** **1:** **Figure S1. a** Distribution of tubes on the phantom plates and (**b**) concentration of solutions in each phantom plate. **Table S1**. Measurements of T_1_ and T_2_ values (mean and standard deviation) obtained for some representative NiCl_2_ and MnCl_2_ solutions.

## Data Availability

Data will be made available on reasonable request.

## Abbreviations

3D: Three-dimensional
CASPER: CASual Phantom for Easy Relaxometry
CSF: Cerebrospinal fluid
GM: Gray matter
MP2RAGE: Magnetization prepared 2 rapid acquisition gradient-echoes
MPRAGE: Magnetization-prepared rapid gradient-echo
MRI: Magnetic resonance imaging
RF: Radiofrequency
ROI: Region of interest
SI: Signal intensity
TSE: Turbo spin-echo
WM: White matter

## References

[1] Baudrexel S, Nürnberger L, Rüb U et al (2010) Quantitative mapping of T1 and T2* discloses nigral and brainstem pathology in early Parkinson’s disease. Neuroimage 51:512–520. 10.1016/j.neuroimage.2010.03.005 20211271 10.1016/j.neuroimage.2010.03.005

[2] Srinivasan R, Henry R, Pelletier D, Nelson S (2003) Standardized, reproducible, high resolution global measurements of T1 relaxation metrics in cases of multiple sclerosis. AJNR Am J Neuroradiol 24:58–67 12533328 PMC8148959

[3] Lindner T, Guerreiro H, Austein F, Fiehler J (2022) Individualized arterial spin labeling background suppression by rapid T1 mapping during acquisition. Eur Radiol 32:4521–4526. 10.1007/s00330-022-08550-8 35118530 10.1007/s00330-022-08550-8 PMC9213266

[4] Fallatah SM, Pizzini FB, Gomez-Anson B et al (2018) A visual quality control scale for clinical arterial spin labeling images. Eur Radiol Exp 2:45. 10.1186/s41747-018-0073-2 30569375 10.1186/s41747-018-0073-2 PMC6300452

[5] Mandine N, Tavernier E, Hülnhagen T et al (2023) Corpus callosum in children with neurodevelopmental delay: MRI standard qualitative assessment versus automatic quantitative analysis. Eur Radiol Exp 7:61. 10.1186/s41747-023-00375-4 37833469 10.1186/s41747-023-00375-4 PMC10575841

[6] Danieli L, Riccitelli GC, Distefano D et al (2019) Brain tumor-enhancement visualization and morphometric assessment: a comparison of MPRAGE, SPACE, and VIBE MRI techniques. AJNR Am J Neuroradiol 40:1140–1148. 10.3174/ajnr.A6096 31221635 10.3174/ajnr.A6096 PMC7048542

[7] Conte GM, Altabella L, Castellano A et al (2019) Comparison of T1 mapping and fixed T1 method for dynamic contrast-enhanced MRI perfusion in brain gliomas. Eur Radiol 29:3467–3479. 10.1007/s00330-019-06122-x 30972545 10.1007/s00330-019-06122-x

[8] Verry C, Dufort S, Lemasson B et al (2020) Targeting brain metastases with ultrasmall theranostic nanoparticles, a first-in-human trial from an MRI perspective. Sci Adv 6:eaay5279. 10.1126/sciadv.aay5279 32832613 10.1126/sciadv.aay5279 PMC7439298

[9] Verry C, Dufort S, Villa J et al (2021) Theranostic AGuIX nanoparticles as radiosensitizer: a phase I, dose-escalation study in patients with multiple brain metastases (NANO-RAD trial). Radiother Oncol 160:159–165. 10.1016/j.radonc.2021.04.021 33961915 10.1016/j.radonc.2021.04.021

[10] Crawley AP, Henkelman RM (1988) A comparison of one-shot and recovery methods in T1 imaging. Magn Reson Med 7:23–34. 10.1002/mrm.1910070104 3386519 10.1002/mrm.1910070104

[11] Look DC, Locker DR (1970) Time saving in measurement of NMR and EPR relaxation times. Rev Sci Instrum 41:250–251. 10.1063/1.1684482

[12] Stikov N, Boudreau M, Levesque IR et al (2015) On the accuracy of T1 mapping: searching for common ground. Magn Reson Med 73:514–522. 10.1002/mrm.25135 24578189 10.1002/mrm.25135

[13] Homer J, Beevers MS (1985) Driven-equilibrium single-pulse observation of T1 relaxation. A reevaluation of a rapid “new” method for determining NMR spin-lattice relaxation times. J Magn Reson 63:287–297. 10.1016/0022-2364(85)90318-X

[14] Marques JP, Kober T, Krueger G et al (2010) MP2RAGE, a self bias-field corrected sequence for improved segmentation and T1-mapping at high field. Neuroimage 49:1271–1281. 10.1016/j.neuroimage.2009.10.002 19819338 10.1016/j.neuroimage.2009.10.002

[15] Thangavel K, Saritas E (2017) Aqueous paramagnetic solutions for MRI phantoms at 3 T: a detailed study on relaxivities. Turkish J Electr Eng Comput Sci 25:2108–2121. 10.3906/elk-1602-123

[16] Lux F, Mignot A, Mowat P et al (2011) Ultrasmall rigid particles as multimodal probes for medical applications. Angew Chem Int Ed Engl 50:12299–12303. 10.1002/anie.201104104 22057640 10.1002/anie.201104104

[17] Hagiwara A, Fujimoto K, Kamagata K et al (2021) Age-related changes in relaxation times, proton density, myelin, and tissue volumes in adult brain analyzed by 2-dimensional quantitative synthetic magnetic resonance imaging. Invest Radiol 56:163–172. 10.1097/RLI.0000000000000720 32858581 10.1097/RLI.0000000000000720 PMC7864648

[18] Lavielle A, Boux F, Deborne J et al (2023) T1 mapping from MPRAGE acquisitions: application to the measurement of the concentration of nanoparticles in tumors for theranostic use. J Magn Reson Imaging 58:313–323. 10.1002/jmri.28509 36315197 10.1002/jmri.28509

[19] Shin W, Gu H, Yang Y (2009) Fast high-resolution T1 mapping using inversion-recovery Look-Locker echo-planar imaging at steady state: optimization for accuracy and reliability. Magn Reson Med 61:899–906. 10.1002/mrm.21836 19195021 10.1002/mrm.21836 PMC2663603

[20] Stupic KF, Ainslie M, Boss MA et al (2021) A standard system phantom for magnetic resonance imaging. Magn Reson Med 86:1194–1211. 10.1002/mrm.28779 33847012 10.1002/mrm.28779 PMC8252537

[21] Bapst B, Amegnizin J-L, Vignaud A et al (2020) Post-contrast 3D T1-weighted TSE MR sequences (SPACE, CUBE, VISTA/BRAINVIEW, isoFSE, 3D MVOX): technical aspects and clinical applications. J Neuroradiol 47:358–368. 10.1016/j.neurad.2020.01.085 32017974 10.1016/j.neurad.2020.01.085

[22] Komada T, Naganawa S, Ogawa H et al (2008) Contrast-enhanced MR imaging of metastatic brain tumor at 3 tesla: utility of T_1_-weighted SPACE compared with 2D spin echo and 3D gradient echo sequence. Magn Reson Med Sci 7:13–21. 10.2463/mrms.7.13 18460844 10.2463/mrms.7.13

[23] Kammer NN, Coppenrath E, Treitl KM et al (2016) Comparison of contrast-enhanced modified T1-weighted 3D TSE black-blood and 3D MP-RAGE sequences for the detection of cerebral metastases and brain tumours. Eur Radiol 26:1818–1825. 10.1007/s00330-015-3975-x 26334511 10.1007/s00330-015-3975-x

[24] de Panafieu A, Lecler A, Goujon A et al (2023) Contrast-enhanced 3D spin echo T1-weighted sequence outperforms 3D gradient echo T1-weighted sequence for the detection of multiple sclerosis lesions on 3.0 T brain MRI. Invest Radiol 58:314–319. 10.1097/RLI.0000000000000937 36729811 10.1097/RLI.0000000000000937

[25] Taheri S, Gasparovic C, Shah NJ, Rosenberg GA (2011) Quantitative measurement of blood-brain barrier permeability in human using dynamic contrast-enhanced MRI with fast T1 mapping. Magn Reson Med 65:1036–1042. 10.1002/mrm.22686 21413067 10.1002/mrm.22686 PMC4950947

