# Supplementary material for: Quantitative brain T1 maps derived from T1-weighted MRI acquisitions: a proof-of-concept study
Source: Eur Radiol Exp. 2024 Oct 8;8:109. doi: 10.1186/s41747-024-00517-2 (PMC11461398; doi:10.1186/s41747-024-00517-2)
Supplement: Supplementary file 1 — Additional file 1: Figure S1. a Distribution of tubes on the phantom plates and (b) concentration of solutions in each phantom plate. Table S1. Measurements of T1 and T2 values (mean and standard deviation) obtained for some representative NiCl2 and MnCl2 solutions. [file 41747_2024_517_MOESM1_ESM.pdf]

Quantitative brain T1 maps derived from T1-weighted MRI acquisitions: a proof-of-concept study

ELECTRONIC SUPPLEMENTARY MATERIAL

a

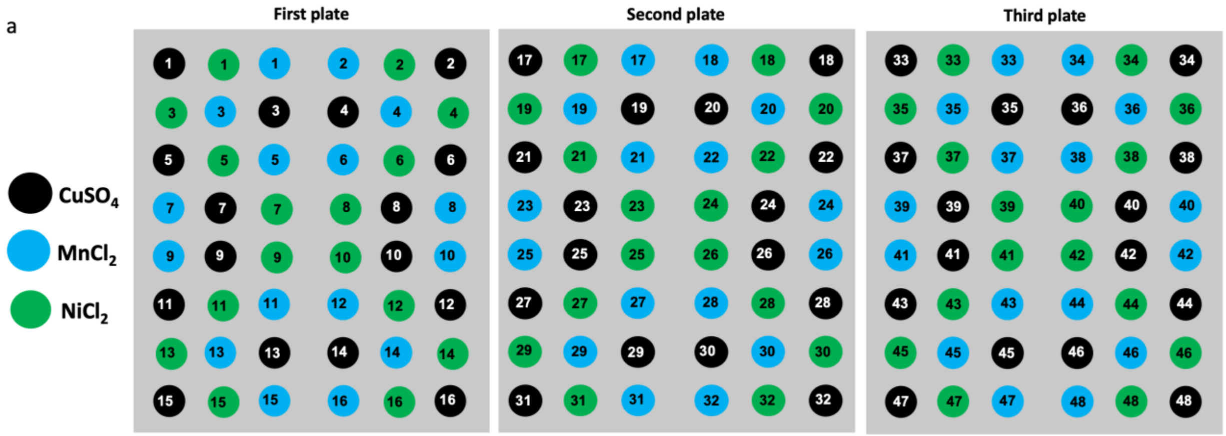

b

	$CuSO_4$	$MnCl_2$	$NiCl_2$
**First plate (1 to 16)**	1 mM	0.002 – 0.152 mM	0.030 – 1.530 MM
**Second plate (17 to 32)**	1 mM	0.162 – 0.312 mM	1.630 – 3.130 mM
**Third plate (33 to 48)**	1 mM	0.322 – 0.472 mM	3.230 – 4.730 mM
**Increment**		0.01 mM	0.1 mM

Figure S1. a. Distribution of tubes on the phantom plates and **b.** concentration of solutions in each phantom plate.

**$MnCl_2$**	**Concentration (mM)**	0.262	0.302	0.342	0.382	0.422	0.462
	**T_1 (s)**	0.378 ± 0.031	0.349 ± 0.028	0.323 ± 0.038	0.290 ± 0.024	0.329 ± 0.020	0.224 ± 0.022
	**T_2 (s)**	0.033 ± 0.001	0.028 ± 0.001	0.025 ± 0.001	0.024 ± 0.002	0.023 ± 0.002	0.021 ± 0.001
**$NiCl_2$**	**Concentration (mM)**	0.730	1.530	2.330	3.130	3.930	4.730
	**T_1 (s)**	1.308 ± 0.036	0.823 ± 0.018	0.640 ± 0.016	0.549 ± 0.012	0.403 ± 0.045	0.362 ± 0.034
	**T_2 (s)**	0.347 ± 0.011	0.249 ± 0.008	0.209 ± 0.004	0.225 ± 0.004	0.155 ± 0.002	0.165 ± 0.002

Table S1. Measurements of T_1 and T_2 values (mean and standard deviation) obtained for some representative $NiCl_2$ and $MnCl_2$ solutions.
